# Beautiful and delicious mutants: The origins, fates, and benefits of molecular sequence variation in plant evolution and breeding

**DOI:** 10.1093/plphys/kiaf378

**Published:** 2025-08-28

**Authors:** Thomas L Slewinski, Sarah Turner-Hissong, Tomasz Paciorek, Brent Brower-Toland, Christine Shyu

**Affiliations:** Bayer U.S. Crop Science, 700 Chesterfield Parkway West, Chesterfield, MO 63017, USA; Bayer U.S. Crop Science, 700 Chesterfield Parkway West, Chesterfield, MO 63017, USA; Bayer U.S. Crop Science, 700 Chesterfield Parkway West, Chesterfield, MO 63017, USA; Bayer U.S. Crop Science, 700 Chesterfield Parkway West, Chesterfield, MO 63017, USA; Bayer U.S. Crop Science, 700 Chesterfield Parkway West, Chesterfield, MO 63017, USA

## Abstract

Heritable sequence changes conferred by mutations have historically been, and continue to be, a valuable source of genetic variation in plant breeding to deliver vegetables, fruits, flowers, and grains with improved quality, diversity, and performance. Genetic diversity in domesticated crops is not entirely preexisting or fixed. This diversity depends on the progression of breeding tools and methodologies that deliver mutations to the enterprise of plant improvement. While breeding has been part of human history for thousands of years, DNA was not recognized as the molecular basis of inheritance until the 1940s. Even more recently, sequencing technologies have allowed us to reveal the allelic variation responsible for naturally occurring phenotypic characteristics that were advanced by evolution and selective breeding. Here, we summarize specific examples of sequence variation that illustrate the extent and impact of plant mutation for agriculture and the essential value of mutational tools to generate additional useful genetic variation. Over time, these tools have been successfully deployed in plant breeding and have been accepted as a means to produce beneficial variation in crops without compromising safety. We then describe the potential utility of genome editing as a versatile technology to introduce beneficial mutations and to enable plant breeding. Compared with other sources of mutation, genome editing satisfies the same safety requirements while also offering technological advancements to improve the performance and quality of crops that our society depends upon.

## Teaser

Genetic mutations that result from spontaneous errors, random induction, or localized genome editing can produce functionally equivalent outcomes that are safe and useful for crop improvement.

## Introduction

**Figure 1.**
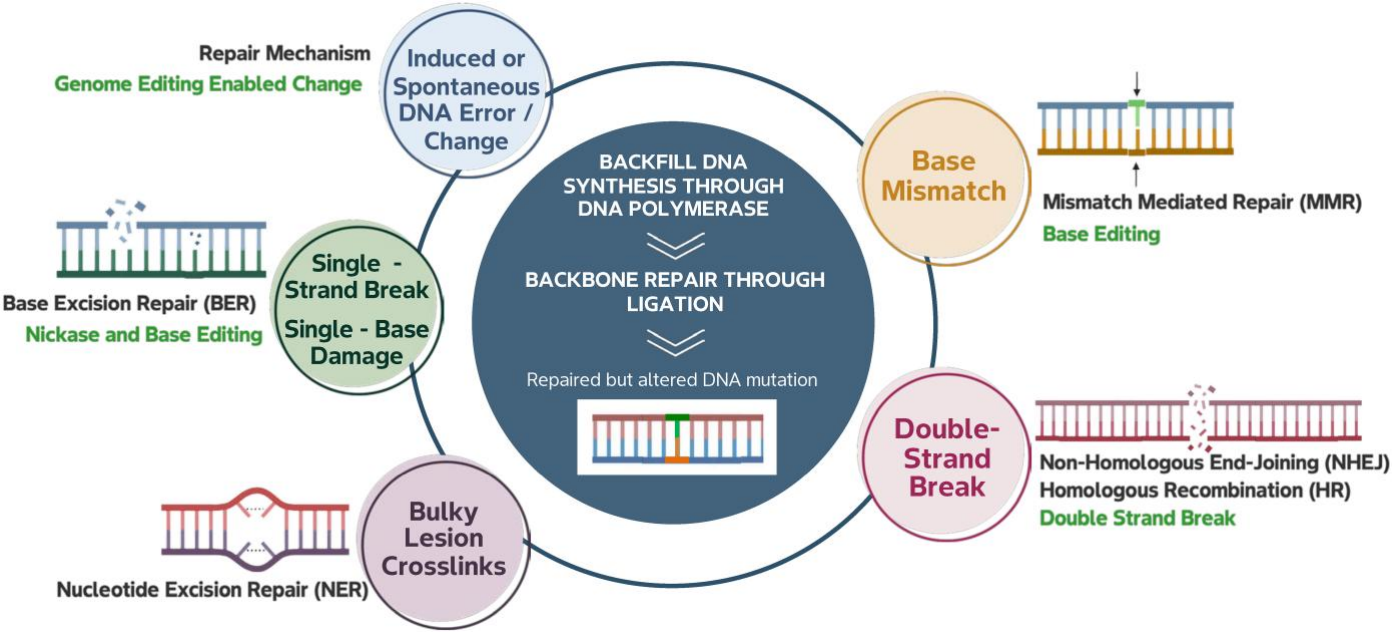
Sources of DNA damage and repair pathways. Sources of DNA damage include cellular errors, biological (e.g. mobile element activity), physical (e.g. UV radiation), or chemical (e.g. EMS) damage. Most DNA damage, whether a base modification or a phosphate backbone interruption, ultimately requires resection, polymerization, and ligation. Genome editing methodologies (highlighted in green text) introduce DNA damage that requires repair through the same endogenous cellular pathways and results in the same types of mutations.

Mutations are changes in DNA sequences that are common in all living organisms, drive phenotypic variation, and reflect heritable sequence changes over time. Throughout history, humans have leveraged mutations alongside favorable recombination events to support crop domestication, diversification, and improvement (Meyer and Purugganan 2013). The mutations that drive plant evolution and selective breeding can occur spontaneously during DNA replication or from exposure to environmental factors like radiation (Fig. 1). Alternatively, mutations can be artificially introduced through methods such as chemical mutagenesis or genome editing. Regardless of how a mutation occurs, the resulting phenotypic variation can be selected for specific traits or advantages based on its impact in a given environmental context (Ahloowalia et al. 2004).

Whether a mutation will persist and rise to high frequency in a population is determined by the mutation rate, selection strength, effective population size, and mating system. The distribution and fate of mutations in populations is well-approximated by the nearly neutral theory of molecular evolution, originally proposed by Tomoko Ohta as an expansion of Motoo Kimura's neutral theory (Ohta 1973, 2002; Kimura 1977). Under this framework, most new mutations are classified as either mildly deleterious or neutral, a smaller fraction as highly deleterious, and the rarest fraction as beneficial. This classification reflects the notion that the most frequent mutations in coding regions are synonymous and do not lead to a change in the resulting amino acid (Kimura 1977). The eventual fate of mutations in each category depends on the strength of selection and population size, with selection exceeding drift when population size is large and the fitness advantage of a mutation is high. Thus, although positive selection has enriched several mutations with large and valuable phenotypic impacts, these beneficial mutations are exceedingly rare, and the vast majority of new mutations are randomly fixed or removed from populations via genetic drift.

In plant breeding, artificial selection aims to promote alleles that enhance traits valued in agriculture, such as changes in plant stature, nutritional quality, and yield, and to remove alleles that confer negative qualities. These characteristics can include fitness-related traits (e.g. yield, biomass, and seed size), but can also include traits that decrease reproductive fitness outside of agricultural settings (e.g. loss of seed shattering). Prior to the availability of nuclease editing technologies in the 1960s, the sources of novel genetic variation available to plant breeders were incidental, relying upon spontaneous mutations or induced random mutations via opportunistic exposure to radiation, chemicals, or biological mutagens like transposons (Gangwar et al. 2025). Given the randomness of these induced mutations, reliance on these methods makes it challenging to acquire the desired frequency and distribution of useful, advantageous mutations in breeding populations.

Over the past decades, the introduction of genome editing tools with targeted mutation methods has provided a more refined means to introduce sequence-specific genetic variation (Box 1). Genome editing tools include technologies like zinc finger nucleases, transcription activator like effector nucleases (TALENs) (Becker and Boch 2021), and clustered regularly interspaced short palindromic repeats (CRISPRs) (Jinek et al. 2012). The ability to introduce targeted sequence changes permits the enrichment of desirable variation while minimizing the introduction of random variation (Graham et al. 2020). As these sequence-specific tools are employed as a source of genetic variation, it is crucial to determine the roles of these methods in existing systems for the generation, modification, understanding, and use of genetic variation in our crop plants.

Box 1. Different mutation sources result in common sequence outcomes. For billions of years, errors in DNA synthesis, exposure to radiation (including sunlight), chemical damage, TEs, and chromosomal damage, with subsequent repair, have been the primary sources of mutations in living organisms (Loewe and Hill 2010). The maintenance and repair of DNA are required at all stages of an organism's life cycle (Brown 2002). These DNA repair mechanisms are required for the integrity of critical cellular reproductive processes like mitosis and meiosis, where DNA is cut and repaired to facilitate recombination. Chromosomal recombination occurs during meiosis, where large portions of homologous DNA can be swapped to assort linked versions (alleles) of genes on a chromosome and give rise to new chromosomal variation in the next generation. Errors can also occur during recombination, leading to the duplication (gene expansion), deletion (gene reduction), or rearrangement of genic regions (Martínez-Fortún et al. 2022). Error rates are usually very low (∼1 × 10^−8^ mutations per base pair per generation in land plants) (Jiang and Ramachandran 2010).

Restriction-based nuclease systems like CRISPR–Cas naturally evolved in prokaryotes as an innovation on the same DNA break and repair mechanisms (Makarova et al. 2020). Genome editing tools, including CRISPR–Cas systems, lead to DNA lesions at targeted locations in the genome that are available to the same repair pathways elicited by spontaneous mutations in the genome (Fig. 1). When-genome editing tools are deployed to edit eukaryotic cells, the resulting mutations to the genome are physically indistinguishable from spontaneous and induced random mutations except that they are targeted to specific locations. Unlike spontaneous and induced mutations, CRISPR–Cas and other genome editing methods are naturally restricted to specific sequences, defined by spacer or guide RNAs in the case of CRISPR–Cas.

In this review, we aim to deconvolute the perception of mutations in plants by discussing genetic sequence changes through 3 lenses: (i) the ubiquity and utility of spontaneous and induced random mutations in agriculturally useful plant varieties, (ii) the importance of mutation as a source of trait variation and yield improvement in plant breeding, and (iii) the potential of genome editing methods to both recapitulate desirable existing mutations and to intentionally introduce new beneficial mutations at specific genome locations. With a more complete understanding of the foundational roles of mutations and genetic variation in agriculture, improved tools and methods can be introduced to meet the needs of a growing population and the challenges of a changing climate.

### The perception of the term “mutation” is context-dependent

Genetic diversity in plants, particularly in domesticated crop varieties, is a valuable resource to support crop improvement and food security, as evidenced by global efforts to catalog and preserve this diversity in germplasm collections (Hoisington et al. 1999). The genetic composition of domesticated plant populations was developed over millennia through a combination of stochastic processes like genetic drift and intentional processes like selective breeding. Intentional selection has enhanced useful characteristics for human societies, providing the raw materials used for construction, nutrition, and medicine today. Genetic mutations in plant genomes were also crucial for the development of crops that are more palatable, less toxic, higher yielding, and more adaptable to regions of human migration and habitation (Meyer and Purugganan 2013; Hufford et al. 2019). Just as natural selection requires mutations to enable speciation over time, artificial selection requires mutations to enable the development of new crop varieties. Mutations provide the useful diversity in breeding populations for the stability and success of global human society, particularly in the context of rapidly changing environmental conditions (Salgotra and Chauhan 2023).

Although mutation is the source of the genetic variation that fuels agricultural improvement, the term “mutation” is more often presented to us with negative connotations in the context of entertainment and healthcare. Mutants are usually portrayed as dangerous creatures in books and films. In the medical sphere, mutations are typically associated with genetic disorders, cancer, and other detrimental health conditions (Gilchrist n.d.). In reality, most mutations are neutral or nearly neutral and have little or no impact on the development or physiological function of an organism. Of the mutations with obvious phenotypic effects, an even smaller subset is useful for driving agricultural improvements.

The term “mutation” is only a meaningful description when used in comparison with a preexisting reference, a process that is subject to ascertainment bias (Merrick et al. 2023). In plants, when enough mutations accumulate and result in significant or distinguishable changes in physical or chemical characteristics compared with a reference, a distinction is made to identify individuals harboring these mutations as a new species, population, landrace, cultivar, or named variety (Blakeney 2012). Reference species, lines, or cultivars, along with any associated sequenced genomes, represent a static snapshot in time of an evolving organism. Any identified change or mutation is described in relation to that timepoint and the collection of individuals, tissues, and cells that were sampled. Sequence differences and mutations are relative and are only meaningful in the context of the comparison to another individual, with the comparator itself also being a mutant or variant when compared with a different reference. Even in cases of clonal and asexual propagation, mutations still occur in the form of somaclonal variation, and the driving force of evolution continues as long as the cells in that organism are alive (Duta-Cornescu et al. 2023). As the genomes of more individuals from agricultural species and their wild relatives are sequenced, the extensive scale and magnitude of genetic diversity among the plant species that comprise our food system become more apparent. The most important finding from these comparisons is that most changes in a genome's sequence do not have profound impacts on the phenotype of the plant. Even large sequence changes most often have small quantitative effects. The genomic context of a mutation also matters: a significant fraction of sequence changes occur in noncoding or intergenic regions and are less likely to have a phenotypic impact (Alonge et al. 2020).

This raises the question, “which variant is the mutation in a given comparison?” Is a mutation only considered in reference to the inferred ancestral state of an organism? What constitutes the true ancestral state? Does it matter? It must be noted that differences between cultivated varieties are often quite modest when considering the scale of variation normally observed between wild and domesticated plant varieties. Even so, breeding tools like genome editing, even at current technical potential, introduce targeted sequence variation that is thousands of times smaller than the variation observed between 2 widely used inbred lines of maize (*Zea mays*) (Lorenzo et al. 2023). It is therefore not clear why induced targeted sequence variation is considered to be a separate regulatory class from the sequence variation found in wild populations and conventional crop breeding programs.

### Variation in plant form and function results from genome modifications at every scale, from single base pair mutations to complex genome expansions

**Figure 2.**
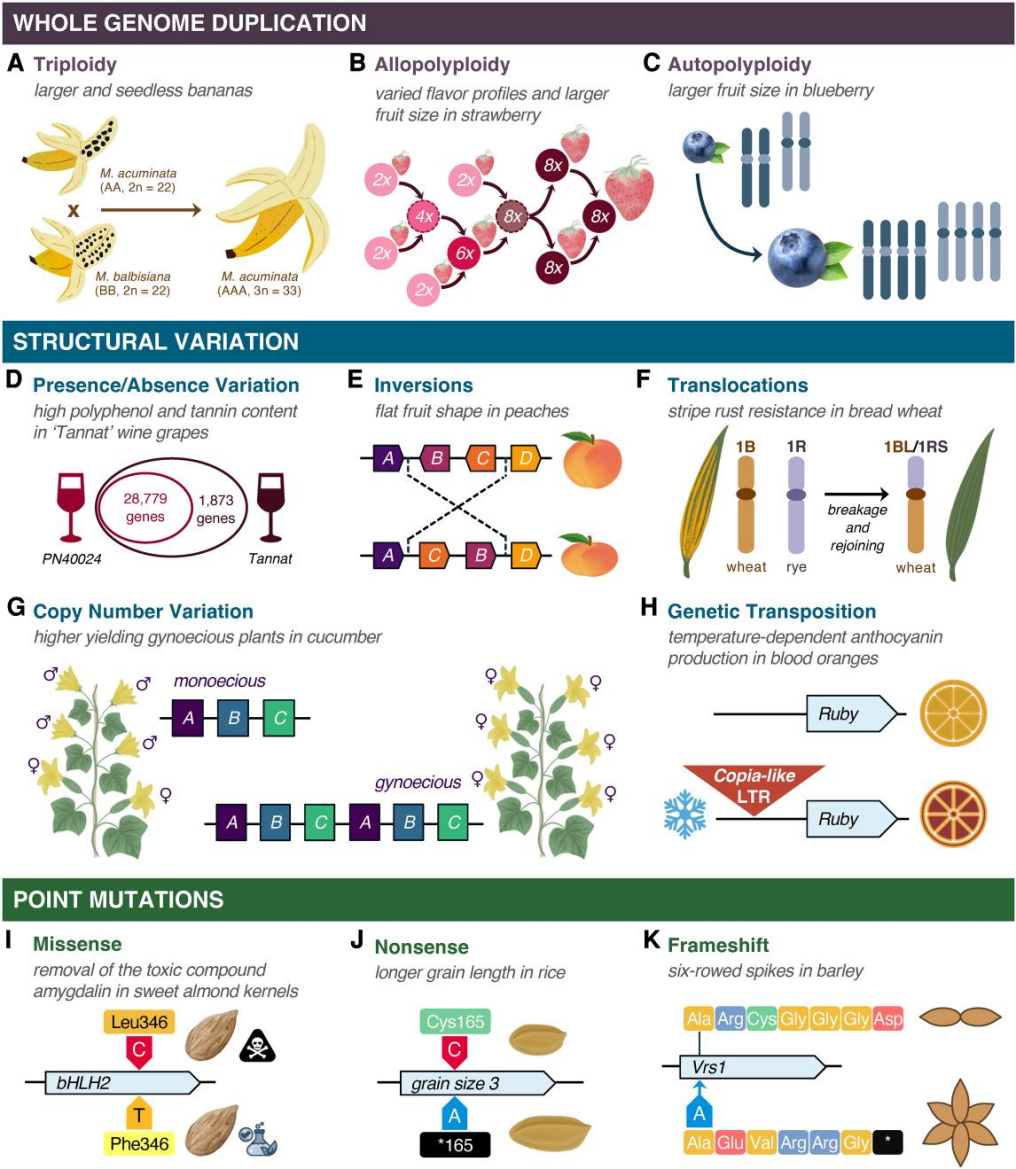
Selective breeding has led to the widespread adoption of naturally occurring sequence modifications with large phenotypic effects, including whole-genome duplications, multiple classes of structural variants, and point mutations. **A)** Seedless bananas (*M. acuminata*, 3*n* = 33) arose from a natural interspecific hybridization between 2 diploid species of banana, *M. acuminata* (2*n* = 22) and *M. balbisiana* (2*n* = 22) (Li et al. 2024). **B)** Octoploid strawberries (*Fragaria × ananassa*) with large fruit size and varied flavor profiles were derived through a complex series of interspecific hybridizations and intermediate polyploids (Bertioli 2019; Edger et al. 2019). Circles indicate ploidy levels and dashed borders indicate an unknown species. **C)** Highbush blueberry (*Vaccinium corymbosum*) is a tetraploid that exhibits larger fruit size compared with diploid wild relatives like Darrow's blueberry (*Vaccinium darrowi*). Recent evidence supports tetrasomic inheritance patterns in highbush blueberry that are consistent with autopolyploidy (Mengist et al. 2023), although there is also evidence supporting allopolyploidy (Colle et al. 2019). **D)** Wines produced from berries of the “Tannat” grapevine cultivar have a unique antioxidant profile and deep purple color. These characteristics were linked to the presence of 1,873 unique varietal genes related to polyphenolic compounds that are absent in the Pinot Noir “PN40024” reference genome (Da Silva et al. 2013). **E)** The compressed shape of flat or “donut” peaches is conferred by a 1.7 Mb inversion that disrupts the expression of an upstream fruit morphology regulatory gene (*Pp Ovate Family Protein1*) (Zhou et al. 2021). **F)** Several yield, disease resistance, and quality traits were integrated into wheat (*T. aestivum*) varieties through interspecific translocations with rye (*S. cereale*), such as the introduction of stripe rust resistance in wheat via a Robertsonian translocation between the long arm of wheat chromosome 1B and the short arm of rye chromosome 1R (Ren et al. 2022). **G)** CNV in a region containing 3 genes at the *femaleness* (*F*) locus in cucumber (*C. sativus*) leads to dosage-dependent gynoecious sex expression, with multiple copies resulting in gynoecious plants that produce only female flowers and have higher yields compared with their monoecious counterparts (Zhang et al. 2015; Li et al. 2020). **H)** The red color of blood oranges results from an insertion of a *Copia*-like LTR retrotransposon in the promoter of the *Ruby* gene that induces anthocyanin production at cold temperatures. **I)** Sweet almonds (*Prunis dulcis*) are nontoxic as the result of a missense mutation that alters the amino acid sequence and resulting protein structure of bHLH2, a transcription factor involved in the biosynthesis of the toxic compound amygdalin (Sánchez-Pérez et al. 2019). **J)** Longer grain length in rice (*O. sativa*) results from a recessive, single nucleotide nonsense mutation that introduces a premature stop codon in the sequence of the *grain size 3* (*GS3*) gene (Takano-Kai et al. 2009). **K)** The conversion from 2-rowed to 6-rowed spikes in barley (*H. vulgare*) results in 3 times the grain production and was traced to multiple, independent loss-of-function mutations at the 6-rowed spike 1 (*Vrs1*) gene. One of the causal mutations, *vrs1.a2*, is a single nucleotide insertion in exon 2 that results in a frameshift of the amino acid sequence (Komatsuda et al. 2007; Casas et al. 2018).

Through their effects on trait variation, sequence modifications at every scale have been effectively and safely selected for crop improvement since the advent of agriculture, despite a lack of knowledge on the underlying genetic changes (Graham et al. 2020). Within plant genomes, mutations range in scale from single nucleotides to the structural rearrangement of larger sequence intervals (deletion, inversion, or transposition), and even the duplication of whole genomes (auto- and allo-polyploidy) (Fig. 2). The landscape of mutations in plant genomes enables the expression of a diverse palette of physical characteristics, contributing to the wide variation of fruit shapes, colors, flavors, and nutritional profiles available in food systems and facilitating rapid evolution in response to environmental selection. Within the context of our food systems, agriculturally significant examples of both spontaneous and induced mutations at every scale demonstrate that mutational tools can lead to the same phenotypic outcomes as naturally occurring sequence variation.

### Whole-genome duplications enable plant adaptation, yield improvement, and innovation

Polyploidy, i.e. when an organism possesses 3 or more sets of homologous or homoeologous chromosomes (Corneillie et al. 2019), is one of the most dramatic and large-scale sequence modifications in plant and animal genomes. Whole-genome duplications occur frequently in the lineages of flowering plants, conferring benefits for plant evolution and agriculture (Fig. 2, A to C). Plants can exhibit stable ploidy levels as high as dodecaploid (2*n* = 12*x*; e.g. some sugarcane species [*Saccharum officinarum*]; the ornamental plumed cockscomb [*Celosia argentea*]) and decosaploid (2*n* = 22*x*; e.g. black mulberry [*Morus nigra*]). Polyploid plants are also classified based on whether multiple sets of chromosomes were derived from the same species (autopolyploids) or from interspecific hybridization (allopolyploids). However, scientific literature demonstrates that the distinction between auto- and allopolyploids is not always straightforward, and the majority of polyploid species are found in a gray area between disomic and polysomic inheritance (Amadeu et al. 2020). Understanding parental heritage becomes especially difficult as plants can undergo several cycles of polyploidization and diploidization over time. Overall, the occurrence of whole-genome duplications is relatively common throughout flowering plant lineages, with an estimated 35% of extant species within genera being recent polyploids (Wood et al. 2009). In addition, many other plant species are ancient polyploids that reverted to a diploid state and benefitted from the outcomes of ancestral genome duplication (Heslop-Harrison et al. 2023).

The evolutionary and agricultural benefits of whole-genome duplication are well studied and include a greater capacity for adaptation to changing environmental conditions, along with a positive correlation between ploidy level and cell size that results in larger leaves, fruits, flowers, seeds, and overall plant size (Otto 2007). In addition to increased organ size, genome dosage confers robustness through heterosis that is realized in many familiar food crops today. Commodities from naturally occurring polyploid crops like wheat (allotetraploid or allohexaploid), sugarcane (*S. officinarum*; allododecaploid), and potato (*Solanum tuberosum*; autotetraploid) are among the top 10 plant-based contributors to global calories per year (FAOSTAT 2022). Triploid plants resulting from hybridization between a diploid parent and a tetraploid parent enable easier processing and consumption of fruit crops by producing seedless varieties in crops such as banana (*Musa acuminata* ssp. *burmannica*) (Li et al. 2024; Fig. 2A) and watermelon (*Citrullus lanatus*; Andrus et al. ). Several commercial fruit crops are also naturally occurring and/or induced polyploids, including kiwifruit (*Actinidia deliciosa*; hexaploid), strawberries (*Fragaria*  *×*  *ananassa*; octoploid), and blueberries (*Vaccinium* sp.; tetra- or hexaploid) (Fig. 2, B and C).

Despite being the consequence of errors in meiosis or mitosis, the agricultural benefits of polyploidy for human society are undeniable. Because whole-genome duplication allows the primary function of a gene to be retained by at least 1 copy in the polyploid genome, the selective pressure on duplicate copies can be lower, permitting diversification among subgenomes through hypo-, sub-, and neo-functionalization, dosage balance, and compensatory drift (Birchler and Yang 2022). This exploration of sequence variation space through accumulated mutations in redundant genes has allowed plants to rapidly adapt to new environments, develop new capabilities, and modify existing chemical synthesis pathways for growth and defense against pests and pathogens (Liu et al. 2017).

### Structural variants influence recombination frequency, sexual dimorphism, and gene regulatory networks to drive biochemical and morphological innovations

The mutant class known as structural variants encompasses larger mutations (sequence changes >50 bp) where a genetic sequence is inserted, deleted, duplicated, translocated, or inverted at a given locus (Fig. 2, D to H). Structural variants in isolation can lead to altered protein sequences, changes in expression, and/or changes in recombination patterns (Alonge et al. 2020). In plants, most structural variation manifests as insertions/deletions (indels) due to translocation or as presence/absence variation (PAV) resulting from nonhomologous recombination (Tao et al. 2019). These structural variants can accumulate over generations, leading to variability in genome size (Gaut and Ross-Ibarra 2008) and amplifying variation among lineages when populations are reproductively isolated (Rieseberg and Willis 2007).

#### Presence/absence variation

In crop species, the extent and impacts of PAV are illustrated by observations of varietal differences in maize and grapevines (*Vitis vinifera*). In maize, a comparison of genome assemblies between the inbred lines Mo17 and B73 found 9,867,466 single nucleotide polymorphisms (SNPs), 1,422,446 indels (<100 bp), and more than 25 Mb of PAV (>500 bp) containing 72 B73-specific PAV genes and 50 Mo17-specific PAV genes (Sun et al. 2018). Among the PAV genes, more than 95% were detected in at least 1 wild relative of maize and ∼25% had likely orthologs in sorghum (*Sorghum bicolor*), suggesting that they may have resulted from the rediploidization of tetraploid ancestors prior to maize domestication. In grapevine, Da Silva et al. (2013) found that “Tannat,” a French-derived cultivar that is grown in Uruguay and is prized for its high quality, vibrant purple color, and composition of phenolic compounds, contains 1,873 genes that are absent in the reference genome of cultivar “PN40024” (Da Silva et al. 2013; Fig. 2D). These 1,873 genes were determined to be cultivar-specific and were enriched for enzymes in the phenylpropanoid and flavonoid pathways. The expression of these genes altered naturally occurring biosynthetic processes so that the production of polyphenols, in particular anthocyanidins, was shunted into branched synthesis pathways, producing the unique chemical profile found in “Tannat” berries and wine.

#### Inversions

Chromosomal inversions occur when a segment of DNA breaks and is reintegrated in the reverse orientation. Due to the rearrangement of the sequence needed for homologous pairing of the chromosome arms, effective recombination can be suppressed in regions containing an inversion (Wellenreuther and Bernatchez 2018; Huang and Rieseberg 2020). This distortion of genetic linkage in regions with inversions affects the ability of selective breeding to incorporate new genetic variation and to achieve specific allele combinations. As a result of this reduced recombination, inversions are often associated with sex-linked regions in hetero- and homo-morphic sex chromosomes across plant and animal lineages (Olito and Abbott 2023). Papaya (*Carica papaya*) is an example of a crop with a recently evolved XY chromosome pair, where 2 Y chromosomes lead to the development of males (Y) and hermaphrodites (Y^h^). Two large inversions in the hermaphrodite-specific regions between the X and Y^h^ chromosomes likely mediated the divergence of sex chromosomes in papaya by suppressing recombination and enabling the subsequent proliferation of other chromosomal rearrangements (Wang et al. 2012b).

In terms of size, inversions in plants are typically large and range from ∼130 kb to 100 Mb, with an average size of 8.4 Mb (Wellenreuther and Bernatchez 2018). As a consequence, inversions often alter the expression of the genes that are adjacent to the inverted region. This is the case in flat peach (*Prunus persica*) varieties, where the flat, disc-like fruit shape results from a 1.7 Mb inversion that alters the expression of *Pp Ovate Family Protein1*, a major regulator of fruit morphology that is located 3 kb upstream of the inversion (Zhou et al. 2021; Fig. 2E).

#### Translocations

Translocations occur when segments of a chromosome break and reattach to a different chromosome, a phenomenon that is particularly common in allopolyploid species due to homeologous exchanges (Mason and Wendel 2020). This reshuffling of chromosomes can be balanced, where there is an equivalent amount of genetic material exchanged, or unbalanced, with a concomitant loss or gain of genes. Several well-characterized translocations have contributed valuable traits to agricultural crops. For instance, global wheat breeding efforts have leveraged a series of lines that carry a Robertsonian translocation between the short arm of chromosome 1 in rye (*Secale cereale*; 1RS) and the long arm of chromosome 1 in wheat (1BL) (Fig. 2F). Genes contained in the 1RS chromosome arm confer resistance to multiple diseases and pests along with greater yield potential (Ren et al. 2022; Han et al. 2025). Another example is a nonreciprocal translocation between regions of chromosomes A05 and C05 in *Brassica napus* that confers lower seed lignin content through the deletion of *PAL4*, a lignin biosynthetic gene (Schilbert et al. 2023).

#### Copy number variation

Copy number variation (CNV) results from deletions or duplications (tandem or nonlocal) of genomic sequences of >50 bp, leading to differences in the number of repeats for that sequence across individuals of a species. In cucumber (*Cucumis sativus*), CNV at the *Female* (*F*) locus leads to dosage-dependent gynoecious plants that only bear female flowers and set fruit at every node (Zhang et al. 2015; Fig. 2G). The causative CNV is a 30.2 kb duplication containing 3 genes at a meiotically unstable region of the genome and most likely arose from an error during microhomology-mediated DNA repair (Zhang et al. 2015; Li et al. 2020).

In sorghum, CNV plays an interesting role in dwarf plant stature, a valuable trait for improved lodging resistance and yield potential. One of the first native mutant alleles identified for the dwarf phenotype in sorghum, *dw3-ref*, contains an 882 bp tandem duplication in the *dwf3* gene that interferes with the function of the corresponding auxin transport protein (Multani et al. 2003). However, fields of dwarf sorghum with the *dwf3-ref* allele had repeated observations of tall, wild-type plants at a rate of 1/600 plants (Karper 1932). It was later discovered that the duplication in the *dwf3-ref* allele leads to unequal crossing-over during meiosis, causing sporadic reversion to the wild-type allele and requiring the identification of alternative mutations at the *dwf3* gene for height stability in dwarf plants (Diatta-Holgate et al. 2024).

#### Genetic transposition

Transposable elements (TEs), or “jumping genes,” are endogenous structural variants that typically range from a 100 bp to 10 kb in length and are major contributors to genome size and phenotypic variation in eukaryotes (Wells and Feschotte 2020). The moniker “jumping genes” refers to the ability of TEs to change position in the genome through excision, insertion, and duplication. The insertion or deletion of TEs into new location(s) can alter the expression of genes by modifying promoter regions, untranslated regions, and introns or can disrupt gene function if inserted into the protein coding regions of a gene (Hirsch and Springer 2017). Notably, TEs are present at relatively high frequencies in many plant genomes, with estimates of TEs comprising 20% of genome content in oranges, melons, and strawberries to over 80% in maize, barley, wheat, and sunflower (Vitte et al. 2014).

The composition, activity, and impact of TEs in plant genomes are determined by a multitude of factors like genomic context and the mechanism of TE activity. Genomic context includes factors like methylation and chromatin state, which influence where a TE can insert in the genome, whether it can replicate/excise, and how it will impact phenotypic variation (Stitzer et al. 2021; Catlin and Josephs 2022). The mechanism of TE activity is largely captured by the categorization of TEs into major classes based on replication and insertion patterns: Class I (retrotransposons, “copy-and-paste” elements) and Class II (DNA transposons, “cut-and-paste” elements) (Wells and Feschotte 2020). Many class II TEs have the unique ability to excise and stitch flanking genomic regions back together. Provided the excision of a TE does not interfere with the coding region, damage an expression element, and/or remove a flanking sequence, this process can restore the genomic location of a TE insertion back to its previous state (Anderson et al. 2019). In cases where a TE insertion influences a phenotype, a subsequent excision event can result in a reversion of this phenotype.

The dynamic, transient, and complex nature of TEs as components of genotypic and phenotypic variation challenges the assertion that mutations must be stable to be useful and safe in agriculture. There are numerous examples of naturally occurring TE insertions that confer valuable characteristics in food crops. Some key examples are flowering time modifications to enable broad geographic adaptation in soybean (*Glycine max*) via retrotransposon insertions at multiple loci (Tsubokura et al. 2014), and, when paired with cold temperatures, higher anthocyanin production in Sicilian blood oranges (*Citrus*  *×*  *sinensis “Blood orange”*) via a *Copia-like* retrotransposon insertion adjacent to the *Ruby* gene (Butelli et al. 2012; Fig. 2H). The transition from a multi-stem architecture to a single stalk during the domestication of maize from teosinte is one of the most important and striking examples of how TEs can create variation. A *Hopscotch-type* Ty1/Copia family retrotransposon insertion into a gene regulatory element over 63 kb upstream of the *teosinte branched 1* gene (*tb1*, encoding a TEOSINTE BRANCHED 1, CYCLOIDEA, PCF1-type transcription factor) led to increased gene expression, suppressed axillary branching, and devoted a greater amount of resources to tassel and ear development (Studer et al. 2011). This shift in plant architecture has allowed maize plants with 1 to 2 large ears per stalk to be cultivated at higher planting densities, setting the foundation for current levels of industrialized maize production.

### Base pair level changes can confer large-effect phenotypic outcomes

Although they occur at the smallest scale on the mutational landscape, point mutations like transitions (purine-to-purine) and transversions (purine-to-pyrimidine or vice versa) have substantially contributed to phenotypic variation and underlie many prominent characteristics in food crops (Merrick et al. 2023). Single base substitutions and small indels are often spontaneously introduced by errors during DNA replication or during DNA repair following exposure to mutagens like UV radiation from sunlight.

Numerous examples of single base pair substitutions highlight their importance in the development of safe, palatable, nutritious foods. A particularly compelling example is the selection of sweet almonds (*Prunus dulcis*) from bitter almonds (*P. dulcis* syn. *Prunus amygdalus*) to enable widespread human consumption. The kernels of bitter almonds are unpalatable, highly toxic, and potentially lethal to humans due to the production of amygdalin, a cyanogenic diglucoside that accumulates in the cotyledons, stems, leaves, and roots of bitter almond (Ladizinsky 1999). Consequently, the initial cultivation of bitter almonds prioritized downstream uses in fragrant oils and extracts alongside the development of processing methods to remove glycosides in bitter almond derivatives like flavorings, a practice that is still ongoing today (Leman 2019). Attempts to reduce the toxicity also occurred through selection for palatability, leading to the key biochemical innovation of reduced amygdalin content in sweet almonds. The underlying sequence change for this biochemical innovation is a dominant, nonsynonymous C to T transition mutation in a basic helix-loop-helix transcription factor gene (*bHLH2*) (Fig. 2I). This mutation interferes with the protein structure of the bHLH2 transcription factor, rendering it unable to induce transcription of the P450 monooxygenase genes required for the biosynthesis of amygdalin (Sánchez-Pérez et al. 2019). While selection on the dominant *bHLH2* mutation produced sweet, palatable almond kernels, lower amygdalin content led to reduced defense capacity against major pests like the buprestid beetle *Capnodis tenebrionis* (Dicenta et al. 2002). The larvae of these beetles burrow into the roots and stems of almond trees and can kill a large tree within 2 yr (Nasouri 2024). To retain the insect-protective properties of amygdalin in roots while maintaining low levels in kernels, sweet almonds are commonly grafted onto bitter almond rootstock (ORGANIC Seeds n.d.).

### Mutations at all scales can produce similar phenotypic outcomes

**Figure 3.**
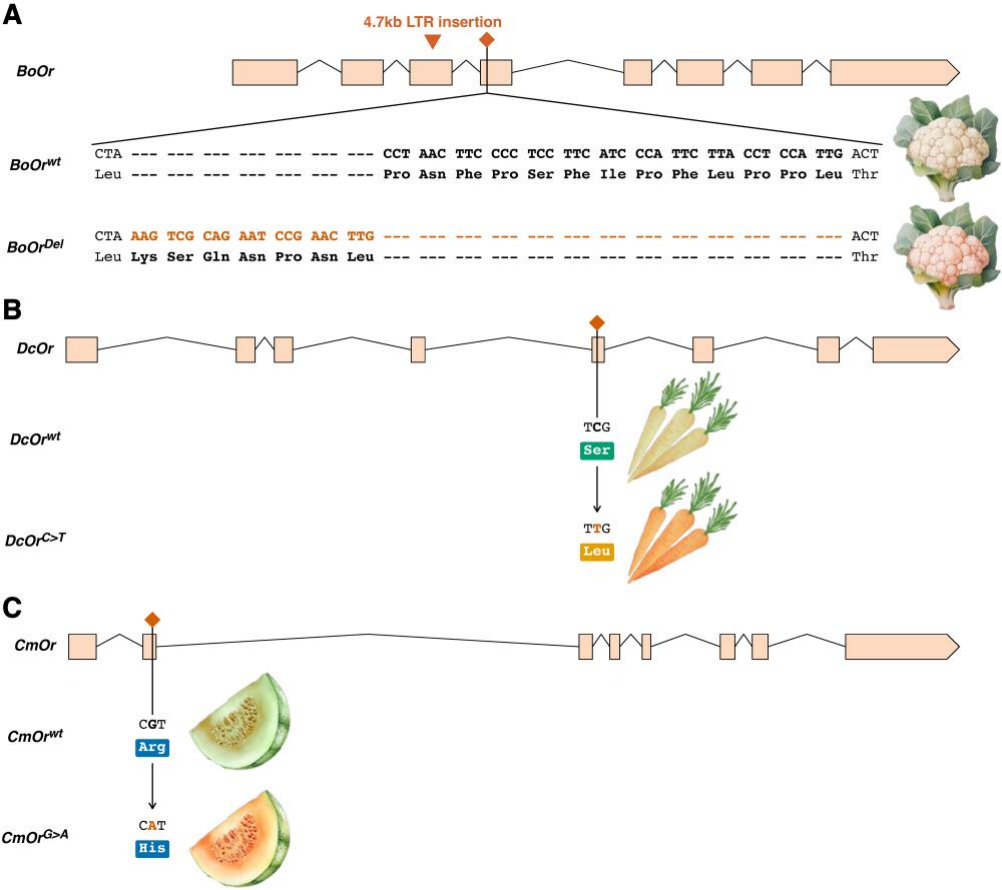
Different mutations can lead to convergent phenotypic outcomes. The high beta-carotene phenotypes in cauliflower, carrot, and melon show that different types of mutations in orthologs of the *Orange* (*Or*) gene can result in convergent functional and phenotypic outcomes within and across species. **A)** A 4.7-kb LTR retrotransposon insertion into the third exon of the *BoOr* gene leads to a splice variant that produces a dominant gain-of-function allele for high beta-carotene in cauliflower (Lu et al. 2006). **B)** Orange carrots carry a recessive single base pair transition (C to T) in exon 5 of the *DcOr* gene, leading to a missense mutation that converts a serine to a leucine (Ellison et al. 2018). **C)** Orange fleshed melons carry a single base pair transition from G to A in exon 2 of the *CmOr* gene, leading to a missense mutation that converts a highly conserved arginine to a histidine (Tzuri et al. 2015).

Multiple types of mutations can result in convergent functional and phenotypic outcomes within and across species (Fig. 3). For example, convergent selection on independent mutations led to the development of varieties with high beta-carotene content in carrot (*Daucus carota*) (Ellison et al. 2018), cauliflower (*Brassica oleracea* var*. botrytis*) (Lu et al. 2006), melon (*Cucumis melo*) (Tzuri et al. 2015), and sweet potato (*Ipomoea batatas*) (Gemenet et al. 2020). Compared with their wild relatives, orange varieties of these crops accumulate higher levels of provitamin A carotenoids like beta-carotene. While there is no apparent selective or evolutionary advantage of higher carotenoid content in the consumed portions of these crops (Yuan et al. 2015), there is a significant benefit for human health by providing a valuable dietary source of provitamin A compounds. Vitamin A deficiency in the early years of childhood development can lead to lifelong physical and health problems including cognitive impairment, developmental delay and, in severe cases, permanent blindness, morbidity, and/or mortality.

At the sequence level, orange varieties of cauliflower, carrot, and melon all contain distinct, gain-of-function mutations in orthologs of the *Orange* (*Or*) gene (Fig. 3). The *Or* gene encodes a cystine-rich plastid-localized DNAJ chaperone protein involved in post-transcriptional regulation of phytoene synthase, one of the rate-limiting enzymes in the carotenoid biosynthetic pathway. This gene also plays significant roles in the formation, division, maturation and function of chromoplasts (plastid-derived organelles that synthesize and store fat-soluble pigments). In the case of cauliflower, the high carotenoid allele arose from a 4.7 kb long terminal repeat (LTR) retrotransposon insertion into the third exon of the *BoOr* gene, leading to a splice variant that produces a dominant gain-of-function allele (Lu et al. 2006; Fig. 3A). In carrot, individuals with high carotenoid levels carry a single base pair transition (C to T) in exon 5 of *DcOr*, a recessive missense mutation that converts serine to leucine in the translated protein (Ellison et al. 2018; Fig. 3B). Similarly in melon, individuals with high carotenoid levels carry a single base pair transition from G to A in exon 2 of the *CmOr* gene, a missense mutation that converts a highly conserved arginine to histidine (Tzuri et al. 2015; Fig. 3C). In sweet potato, the causal mutation has not yet been isolated, possibly due to the hexaploid nature of the crop (Park et al. 2016), but an ortholog of the *Or* gene is contained within a major quantitative trait locus (QTL) for carotenoid levels in sweet potato tubers (Gemenet et al. 2020).

The above examples of mutations in *Or* genes causing high carotenoid accumulation illustrate the idea that both very small mutations, including single base pair transitions, and relatively large sequence changes like retrotransposon insertions can lead to a similar, desirable outcome in homologous genes across different species. A number of other parallel phenotypes across crops result from diverse mutations in orthologous genes, including shattering resistance in domesticated cereal crop species (Maity et al. 2021).

### Mutations act in concert to influence phenotypic variation

The mutational landscape of plants does not occur in a vacuum: each individual plant contains a unique constellation of mutations in its genome. The underlying genetic architecture of traits can be due to a single gene (monogenic), a few genes (oligogenic), or many genes (polygenic). Furthermore, epistasis, or nonlinear interactions between or among loci that influence the same trait, can influence the impact of alleles in different genetic backgrounds (Mackay and Anholt 2024). Many examples in maize capture the often polygenic and pleiotropic nature of agriculturally relevant traits. For instance, 74 loci were significantly associated with kernel oil concentration and fatty acid composition in maize kernels. Among these 74 loci, 26 loci associated with oil concentration could explain up to 83% of the phenotypic variation using a simple additive model (Li et al. 2013). Another example is plant architecture in maize, which affects planting density in the field. For phenotypic characteristics including leaf angle, leaf length, leaf width, and days to silking, over 30 QTLs contribute to each characteristic with very little overlap (Tian et al. 2011). Looking holistically at genetic improvement during modern maize breeding, (Wang et al. 2020) identified 160 loci underlying adaptive agronomic phenotypes and more than 1,800 genomic regions representing the targets of selection during modern breeding based on an analysis of 350 elite inbred lines representing germplasm from China and the United States.

Tomato (*Solanum lycopersicum*) fruit size and shape is another example of how a combination of mutation types can lead to differences that underpin market classes in a food crop (Sun et al. 2017). At the molecular level, tomato fruit size is determined by how many locules it develops, a trait that is primarily controlled by 3 genetic loci: fasciated (*FAS*) (Cong et al. 2008), number of floral organs (*ENO*) (Yuste-Lisbona et al. 2020), and locule number (*LC*) (Muños et al. 2011). The tomato *FAS* gene encodes a CLAVATA3 signaling peptide that acts as a negative regulator of meristem growth and size and also affects fruit development. The mutant *SlCLV3* (*fas*) allele contains an inversion that leads to partial loss-of-function and lower gene expression. Individuals that carry the *fas* mutation produce moderately larger fruit with more locules than the wild type. The *LC* locus contains the *WUSCHEL* gene, a positive regulator of meristem growth and development (Muños et al. 2011). Two SNPs in a putative repression binding site downstream of the *WUSCHEL* gene lead to larger fruits with more locules. The *ENO* locus encodes an AP2/ERF transcription factor involved in regulating meristem activity. The mutant allele contains an 85-bp deletion in its promoter, leading to lower expression of the gene and larger fruit with more locules (Yuste-Lisbona et al. 2020).

Tomato fruit shape is determined by 2 additional loci that control fruit elongation (Rodríguez et al. 2011): *SUN* (Xiao et al. 2008; Jiang et al. 2009) and *OVATE* (Liu et al. 2002). The *SUN* gene encodes a positive regulator of fruit elongation. A *Rider* retrotransposon-induced gene duplication additively increases the expression of the *SUN* gene, leading to increased fruit length. The *OVATE* gene, on the other hand, encodes a negative regulator of growth that functions by repressing the transcription of other growth regulators. A mutation that gives rise to a premature stop codon in the *OVATE* gene leads to a presumed null allele that increases the transcription of genes in the associated target growth pathways (Liu et al. 2002).

In both inbred and hybrid crops, genetic variations in the form of mutations act in concert to influence phenotypic variation. Most quantitative traits are not a result of single changes, but rather, a network of pleiotropic and epistatic molecular changes and interactions that result in the beautiful phenotypic diversity that is observed in nature and selected through plant breeding.

### Random and targeted mutagenesis are valuable tools to accelerate plant breeding

The identification of useful mutations at high resolution is possible through methods that leverage intra- and/or interspecies variation, such as linkage mapping, association studies, pangenomics, and large language models (Wallace et al. 2018). In a breeding program, a beneficial mutation can only have an impact if the individual carrying the mutation is selected and advanced to the next generation. The probability of recovering a favorable recombination or mutation can be improved using large population sizes. However, resources are finite, and increasing population sizes is not a viable strategy to support long-term genetic gain.

In addition to relying on rare beneficial mutations, achieving an optimal combination of alleles through conventional breeding methods is time intensive and confounded by linkage. As a result, it can be challenging to integrate beneficial mutations into new varieties for deployment in large-scale agriculture. For example, if a valuable allele for pest resistance is identified in a wild or distant relative of a crop species, this allele must be introgressed into a desired genetic background via hybridization and backcrossing to achieve the desired outcome: a small portion of a sequence (i.e. the pest resistance allele) moved from 1 genetic background into another. Assuming the absence of hybridization barriers, the process of introgression depends on the cycle time of a given crop, requires multiple years of selective breeding, and introduces unwanted genetic diversity, i.e. linkage drag, from the donor parent. Although technologies such as marker assisted selection have improved the efficiency and precision of introgression, breeding for the optimal combination of mutations that are beneficial to agriculture remains a time intensive and complex process.

Ultimately, plant breeding strategy seeks to maximize the response to selection by minimizing the cycle time and maintaining genetic diversity in breeding populations (Box 2). However, the range of genetic diversity has both direct and indirect limits imposed by founder effects, genetic drift, and intensive selection pressure. From a breeding perspective, mutations and sequence variants are critical wellsprings of genetic and phenotypic diversity to confer higher yields, resilience to changing environments, resistance to pests and diseases, and varied options for consumers. The maintenance of this genetic diversity supports the continuous, long-term, multi-stage processes of domestication and breeding (Hufford et al. 2019; Purugganan 2019, 2022; Huang et al. 2022). Once essential traits (such as nonshattering in grasses and the removal of toxicity in almonds) are achieved through selective breeding, more subtle combinations of sequence variation are necessary to support continued improvements in crop performance, quality, and local adaptation. To maintain pace with the demands for crop performance in changing environments, solutions are needed beyond relying on rare events and time-intensive processes like introgression. Tools such as random and targeted induced mutagenesis increase the probability of introducing beneficial mutations with the potential to reduce the time needed for introgression.

Box 2. Technical barriers limit broad adoption of genome editing. While editing is a promising breeding tool that has led to many advancements, several technical barriers currently limit large-scale deployment.

**1. Delivery of genome editing reagents and regeneration of edited plants**

 Common methods to introduce genome editing reagents include *Agrobacterium* transfection, particle bombardment, and virus-based delivery systems (reviewed in Mao et al. 2019; Nasti and Voytas 2021; Cardi et al. 2023; Bélanger et al. 2024). Many plant species lack established and/or efficient delivery systems to introduce editing machinery into cells. Regeneration methods are also often restricted to certain genetic backgrounds within a species, few of which are commercially relevant.

**2. Determination of editing targets**

 Knowledge of gene function and its association with the desired phenotypic outcome is key to designing impactful editing approaches. Identifying candidate genes requires access to germplasm, genotypic and phenotypic data, and analytical methods. The characterization of specific genes or genomic regions is often done in model species, and translating these findings to crop systems can be challenging (Roeder et al. 2025). Defining exact target regions for editing in relevant plant species often requires additional experimentation and validation.

**3. Ability to obtain edit outcomes efficiently**

 Production of heritable edits requires designing efficient gRNAs for a target sequence. The accessibility of target sites to the editing machinery varies depending on surrounding genomic features, such as methylation status, chromatin state, and the presence of an on-target protospacer adjacent motif that can be recognized by the editing enzyme (Weiss et al. 2022).

 Genome editing can generate an allelic series or a precise edit that recreates a known allelic variant in a different genetic background. Technologies like base pair editors, prime editing, and templated editing expand the horizon of possibilities for precise edits, but require additional development to achieve the efficiency and scale necessary for widespread use in plants (Mao et al. 2019; Bhuyan et al. 2023; Gilbertson et al. 2025).

**4. Scaling to screen edited alleles in breeding populations**

 Complex interactions often occur between edited allele(s) and genetic background(s), posing challenges for the scalability of genome editing in plant breeding. Targeting gene networks, gene families, and/or quantitative traits will likely require editing at many distinct loci (Abdelrahman et al. 2021). To adequately estimate the effects and interactions of edited alleles, the number of plants that must be screened increases dramatically with increasing numbers of target genes. For a more in-depth discussion, see Lorenzo et al. (2023).

#### Induced random mutagenesis

To increase the frequency of genetic and phenotypic variation, plant breeders have implemented mutation breeding, ultimately compressing a millennium-long process into a few years. Mutations can be induced artificially in plants using physical irradiation (e.g. gamma rays, X rays, fast neutrons) or treatment with chemical mutagens (e.g. hydroxylamine, ethylene methyl sulfoxide [EMS]) (Oladosu et al. 2016) or induced using high-activity transposon lines (May et al. 2003). Induced mutagenesis also leverages the DNA repair process to introduce mutations through exposure to ionizing radiation or chemical mutagens like EMS (Till et al. 2004). It is critical to emphasize that the production of new mutations through these processes will not persist once the induction agent is removed or degrades. To date, random mutation breeding has led to the development of at least 3,640 plant varieties that have added novel variation to grains, vegetables, fruits, and flowers across the globe (FAO/IAEA n.d.). Many of these mutant varieties are directly mutagenized cultivars or lines with improved agronomic and botanical traits, abiotic stress tolerance or resistance, or better nutritional or quality traits (examples in Table 1; Sebastian et al. 2023). Importantly, in almost all cases, the specific sequence changes in the mutant that give rise to the different phenotypes and characteristics are not described, and their description is not required for the release of a new variety.

**Table 1.** Examples of released and named mutant varieties from the “Mutant Variety Database”

Species	Variety name	MVID	Year of release	Mutant characteristics	References	Country of origin
Onion (*Allium cepa*)	“Compas” “Brunette”	2227 2226	1970 1973	More dry layers for long keeping and mechanical harvesting High yield and early maturing	Sigurbjoernsson and Micke (1974)	Netherlands
Clementine (*Citrus clementina*)	“Nero” “Clemenverd” “Neufina”	3450 3437 3451	2006 2010 2009	Early maturing, large fruit and seedless Late maturing, large fruit and seedless Fruit retention, easy to peel and seedless	Fresh Plaza (2013)	Spain
Lemon (*Citrus limon* L. Burm.)	“Eylul” “Alata” “Gulsen” “Uzun”	4949 4948 4950 4951	2009 2010 2010 2010	Early maturing and disease resistance Seedless Seedless Seedless	Uzun et al. (2008)	Turkey
Taro (*Colocasia esculenta*)	“Chiba-maru”	2901	2007	Round tubers	Suzuki et al. (2006)	Japan
Sweet Potato (*Ipomoea batatas*)	“Yushu 5”	2244	1990	Higher yielding, more medium sized tubers	Xiu-Zhen (1990)	China
Lettuce (*Lactuca sativa*)	“Ice cube” “Blush” “Mini-green”	2229 2230 2231	1992 1992 1992	Dwarf Dwarf Dwarf	Waycott and Ryder (1994)	United States of America
Banana (*Musa* var.)	“Al-Beely”	3404	2007	Higher yielding and stress tolerance	IAEA (2014)	Sudan
Sweet Cherry (*Prunus avium*)	“Stella”	254	1968	Improved self-fertility and cooking/eating quality	Sigurbjoernsson and Micke (1974)	Canada
Rose (*Rosa* sp.)	“Flamingo Queen”	1770	1976	Salmon-pink flower color	Library and Archives Canada (1971)	Canada

The underlying mutations that lead to the characteristics of these varieties are currently unknown.

MVID, Mutant Variety Database Identification Number.

The modern history of plant breeding has demonstrated that the introduction of new genetic diversity through random mutagenesis dramatically accelerates the availability of useful varieties (Yali and Mitiku 2022). Inducing mutations at random locations in the plant genome using ionizing radiation or chemical mutagens creates new alleles that could have occurred in nature but are not frequent enough for humans to identify or are not present in cultivated plant populations and their contemporary wild relatives. The global development of induced mutant varieties began in the 1960s (Ma et al. 2021). Most commercial varieties are from grain crops including rice (*Oryza sativa*), wheat (*Triticum aestivum*), sorghum, barley (*Hordeum vulgare*), and oat (*Avena sativa*), legumes such as chickpea (*Cicer arietinum*) and lentil (*Lens culinaris* subsp. *culinaris*), and floricultural crops such as chrysanthemum (*Chrysanthemum × morifolium*), rose (*Rosa* sp.), alstroemeria (*Alstroemeria aurea*), and begonia (*Begonia* sp.) (IAEA 2022). Varieties that are derived from induced random mutagenesis have a long history of safe use and improved traits that bring additional value, efficiency, and nutrition for growers and the public; this success drives the demand for these new varieties (EFSA Panel on Genetically Modified Organisms (GMO) et al. 2021). Valuable and widely available induced mutations are diverse in structure, ranging from entire genome duplications and chromosomal rearrangements to simple sequence deletions and single base pair changes, as well as insertional transposon-induced mutations.

#### Induced targeted mutagenesis via genome editing

Genome editing is a variation-creating tool that uses nucleases (e.g. TALENs, CRISPR–Cas) to generate targeted alleles that are structurally similar to those obtained in nature or by existing random mutagenesis methods but with precise and refined target sequences (Sharma et al. 2024). Genome editing tools like the CRISPR–Cas system can be introduced into plant cells through transgenes that are subsequently segregated out of edited plants or through meta-stable ribonucleoprotein complexes, which do not require an intermediate integration of the editing machinery into the plant genome; the editing machinery degrades after the edit(s) is/are made (Zhang et al. 2021). These approaches are similar to chemical or radiation-induced mutagenic activities in that editing activity does not persist when the editing machinery is removed. Breeding innovations employing editing nucleases offer a faster, more deliberate, and more efficient way of introducing diversity into agricultural, horticultural, and botanical crops at a time when additional crop diversity is needed to meet global challenges of a rising human population, to deliver sustainable solutions for a changing climate, and to support the conservation of biodiversity.

Targeted mutagenesis of crop plants using editing nucleases offers the potential to develop solutions in principle rather than waiting for the serendipity of rare mutational events and selective conditions. Starting from an understanding of the useful variation that is needed, it is possible to direct mutagenesis, using targeted mutagens like editing enzymes, to introduce mutations at specific genomic targets and create beneficial trait variation (Dudley et al. 2022). In this way, breeding methods like genome editing offer the ability to localize useful variation in genetic regions that are more likely to be productive (Pacher and Puchta 2017).

The value of targeted variation is exemplified by the case of sweet almonds. The *bHLH2* mutation in sweet almonds renders almond kernels palatable and safe for human consumption (Sánchez-Pérez et al. 2019). However, the *bHLH2* mutation is also deleterious for plant fitness by diminishing defense against pests (Dicenta et al. 2002). Targeted breeding technologies like genome editing could be applied to make more specific mutations in *bHLH2* that only reduce amygdalin levels in the kernels while maintaining expression in the roots and other parts of the plant, thereby reducing the potential for negative yield effects from pest damage. This could also be optimized by making multiple changes to the almond genome to optimize the amygdalin pathway in concert with (or even without) the *bHLH2* locus.

While genome editing is a promising tool for crop improvement, it is important to acknowledge that no singular technology is a panacea for the many challenges facing the future of agricultural productivity and sustainability. Continued improvements in genome editing technology and societal acceptance are necessary to realize the value of genome editing as a true breeding tool, particularly across plant species. Key technical challenges and current limitations of genome editing for plant improvement are summarized in Box 3. These challenges are well illustrated by the example of amygdalin production in bitter and sweet almonds, where several limitations prevent the application of genome editing to develop a sweet almond that only produces amygdalin in vegetative tissues like roots and leaves to prevent damage from pests like buprestid beetles. When considering the desired allelic outcomes from editing, it is technically possible to achieve a tissue-specific knockout of amygdalin production in above ground and reproductive tissues while maintaining root specific amygdalin expression and production. However, almonds are recalcitrant to standard transformation and regeneration protocols (Jedličková et al. 2024) and the precise mutation(s) required to achieve the desired tissue-specific expression pattern is difficult to predict from sequence alone and will likely require the testing of multiple alleles to obtain desired outcomes.

Box 3. Precision breeding can increase selection efficacy. The plant breeding process enables short-term evolution via directional, artificial selection toward increased productivity and other beneficial traits. Advancements in breeding methodology seek to improve both the efficacy and efficiency of this process, reducing costs and the time required to develop new varieties (Wallace et al. 2018; Bernardo 2021). The breeder's equation captures how a population responds to selection on a trait and provides a framework for improving genetic gain (Falconer and Mackay 1996). Core components of the breeder's equation include response to selection or the rate of genetic gain (*ΔR*), selection intensity (*i*), selection accuracy (*r*), additive genetic variation (*σ_A_*), and generation interval or cycle time (*L*):

$$\Delta R=\frac{ir\sigma_{A}}{L}$$

Breeding strategies aim to enable long-term genetic gain by decreasing cycle time (*L*), optimizing the accuracy of selection (*r*), and maintaining useful genetic variation (*σ_A_*). Decreases in cycle time are achieved through innovations like genomic selection that identify promising lines quickly (Heffner et al. 2009), 2-part breeding strategies that leverage rapid population improvement and optimal cross design for product development (Gaynor et al. 2017), and methods like speed breeding that increase the number of selection cycles per year (Watson et al. 2018). Improvements in selection accuracy (*r*) have also been achieved through advances in genomic selection models and the development of high-throughput, cost-effective methods for phenotyping and genotyping. While increasing selection intensity (*i*) can promote short-term genetic gain, this strategy risks stagnating the potential for long-term genetic gain by depleting genetic diversity. In all scenarios, breeding strategies must be coupled with the maintenance and continued influx of genetic diversity in breeding populations.

Precision breeding leverages large-scale, directed genome editing to improve the rate of genetic gain by promoting useful genetic variation (*σ_A_*) through the targeted introduction of existing optimal alleles, novel alleles, and novel allelic combinations. Genome editing complements other breeding strategies by enabling the introduction of beneficial alleles at genomic loci associated with traits of interest. Compared with strategies like backcross-mediated introgression that achieve similar outcomes on the levels of trait variation, genome editing can introduce positive variation, bypass the negative consequences of linkage drag, and require fewer cycles to realize genetic gain in population or product development. At the population level, genome editing can increase the efficacy of selection at multiple loci by mitigating phenomena like Hill–Robertson interference, where selection for a beneficial allele can reduce the probability that another beneficial allele reaches fixation (Wallace et al. 2018).

The examples of mutations causing high carotenoid accumulation also demonstrate that both very simple mutations such as single base pair transitions as well as a large 4.7-kb retrotransposon insertion can lead to similar desirable outcomes in homologous genes across different species. Similarly, shattering resistance in domesticated crop species is a result of distinct mutations in homologous genes that confer the same phenotype. Convergent phenotypes across species often harbor different mutations that are functionally interchangeable, despite the source of the causal mutation. Such outcomes could be replicated with genome editing by leveraging homology to introduce functionally equivalent mutations. As a method, genome editing is similar to chemical or radiation-induced mutation (Graham et al. 2020), in that the mutagen is transient and completely absent in the final line or variety. The types of mutations resulting from spontaneous mutation, chemical/radiation-induced mutagenesis, and genome editing as breeding methods are expected to be the same (Box 1). This leads to the question: If the new plant breeding method of genome editing can create base pair variations smaller than a large insertion/deletion and with similar allele structures to random mutagenesis, why would we treat plants developed using this breeding technology any differently? If all mutational outcomes are similar or even identical, the answer to that question is not in the DNA sequence. It is instead related to bias regarding the method used for mutagenesis.

## Genome editing localizes variation to desired regions

The examples of spontaneous mutations described above illustrate how natural and induced genetic variation can lead to changes needed to make plants more nutritious and productive. However, such variation is typically rare and hard to identify when it occurs, thereby limiting the availability of useful mutations in breeding programs. The phenotypic advantages offered by mutant alleles may also come with caveats. Either the phenotypic effect is not of the optimal magnitude, there are additional effects that are detrimental (as in the case of sweet almond losing insect resistance in the root systems), or the trait is not stable (as seen in the case of dwarf sorghum). Intensive selection on useful mutant phenotypes, from both an evolutionary and breeding perspective, can also lead to domestication bottlenecks and founder effects where a subset of individuals from a larger population is overrepresented in the genetic composition of modern breeding populations. Similarly, introgression of genetic diversity from other sources, such as wild relatives, can lead to linkage drag by introducing unwanted genetic variation from the donor parent. Although it may seem contradictory, the introduction of useful large-effect variation into a crop species can lead to a drastic reduction in variation across the genomes of cultivated varieties for that crop. In other words, the spontaneous variants delivered to farmers and breeders by natural mechanisms over thousands of years were usually the only available option, despite potential shortcomings. Since mutant alleles are sometimes imperfect for the selected trait and/or come with trade-offs, additional selection through breeding is often necessary to compensate for these limitations.

The introduction of induced random mutations into breeding programs has alleviated some of the constraints associated with the use of spontaneous variation. However, this conventional process still relies largely on serendipity since the sheer size of plant genomes complicates the identification of the location of induced changes, requiring time and luck to determine where and what changes occurred. Additionally, screening for induced changes requires the growth and evaluation of plants on a large scale (thousands of genotypes) to identify the small fraction of useful genotypes, requiring a huge footprint of economic and physical resources as well as human effort. Unlike conventional mutation breeding, contemporary breeding methods like genome editing are targeted in nature. Localized mutations can be made in known fractions of the crop genome where they are predicted to have the greatest efficacy. This saves time, enables the efficient use of resources, and reduces carbon footprint without inhibiting breeding advances and genetic gain for a given crop. Such economy is particularly valuable where it enables mutation breeding for a crop that would be intractable if random mutagenesis were employed (e.g. perennial or tree crops such as apples, citrus, or grapes). In practice, genome editing can enable breeders to rely less on random chance for the discovery of useful variation and instead focus their resources on specific genomic locations with a higher likelihood of success.

## Safety of mutant selection and breeding

As noted in previous sections of this review, crop domestication and conventional breeding methods depend on natural genetic variation resulting from spontaneous mutations occurring in nature, as well as from randomly induced changes using mutagenic agents. Conventional breeding techniques can significantly alter the genetic makeup of a crop, as demonstrated by the examples in Fig. 2. In commercial breeding programs, extensive plant selection practices are implemented by breeders to remove any unwanted phenotypes and ensure the selection of desired candidates (Glenn et al. 2017). Throughout the years, the collective outcomes of the global crop breeding programs employing these practices have produced new varieties with improved performance and characteristics that constitute most food crops found in grocery stores. The crops derived from conventional breeding methods have not been found to produce harmful novel compounds such as novel toxins (Weber et al. 2012; Steiner et al. 2013; Kaiser et al. 2020), have been safely consumed by humans and animals, and have been safely grown in the environment (Kaiser et al. 2020).

In cases where a crop naturally produces endogenous toxins and antinutritional compounds, additional safety checkpoints are applied (Glenn et al. 2017). For example, new almond varieties developed through conventional breeding are screened by breeders for amygdalin to ensure their content is within limits known to be safe for consumption before a variety is released into the market. Regardless of the breeding method used for line or cultivar development, these crops are carefully evaluated to ensure that the natural levels of toxins or anti-nutrients do not exceed acceptable levels in the consumed parts of the plant.

Global regulatory agencies have recognized the safe history of conventional mutation breeding technologies (Health Canada 2006; EFSA Panel on Genetically Modified Organisms (GMO) 2012; Human Foods Program 2024). Compared with conventional mutation breeding, genome editing technologies create the same types of sequence variation (Fig. 1). Importantly, cultivars and lines developed using genome editing will follow the same standards of practice to ensure safe products for consumers and the environment. Nevertheless, different regulatory oversight standards currently apply to crops developed using conventional breeding methods vs. genome editing technologies, even if they result in crops with the same or similar mutations, see Outstanding questions (Jenkins et al. 2023).

## Conclusions and path forward

Humans have used natural processes to improve plants since our hunter–gatherer ancestors began to plant seeds saved from their preferred fruits, vegetables, and grains. Isolating and harnessing plant phenotypic variation through mutations has been a driving force for agriculture in the co-evolution of humans and their preferred crops (Fuller et al. 2023). The development of landraces and elite varieties today still relies on and benefits from spontaneous and induced mutations. The original domestication of crops did not require underlying knowledge of genes, their modes of action, or the molecular changes that comprise preferred alleles. With innovations in phenotyping and greater accessibility to large-scale sequence data, we now have a much greater awareness of comparative genomics, the underlying genetics of phenotypic variation, and the physiological basis of plant variation. The promise of these advances in agriculture is further realized by the potential of genome editing to modify, combine, and introduce beneficial alleles in breeding pipelines.

There are currently some advocacy groups that often use the terms “freak” or “unnatural” when describing mutations in plants (GMWATCH 2024). These terms are applied to mutations regardless of whether they occur spontaneously through replication error or transposition, are induced through random mutagenesis, or are introduced by targeted mutagenic methods like genome editing. For horticultural crops, the mutations that these advocacy groups define as “freak” are typically termed “beautiful” by the general public (Kayalvizhi et al. 2020; Vishwanath et al. 2024). For example, the roses (*Rosa* spp.) pruned from our garden or purchased from the florist are derived from an array of spontaneous and chemical or radiation-induced mutations that produce the combination of colors, shapes, stems, vase life, thorn density, and flowering time required to achieve the pinnacle of rose beauty (Zhang et al. 2024). By contrast, many wild relatives of rose are considered weeds by farmers and the general public. The rose is only 1 example of the extraordinary power of mutation breeding in the selection of desirable varieties. Yet another striking example is found in the morphotype diversity of vegetables in *B. oleracea*. Given their dramatically distinct appearances, it is often a source of surprise to learn that kale, kohlrabi, cabbage, broccoli, cauliflower, and Brussels sprouts are all members of the same species. This wide array of vegetable types in *B. oleracea* that we admire and enjoy today resulted from artificial selection on naturally occurring mutations to improve palatability and modify appearance. Many of these traits are connected to sequence modifications in developmental pathways, such as the diversification of leaf shapes and textures (e.g. curly and lacinato kales) (Arias et al. 2021), the formation of heads through leaf curling (e.g. cabbage) (Cheng et al. 2016), or alterations of inflorescence morphology (e.g. broccoli and cauliflower) (Lowman and Purugganan 1999; Wang et al. 2012a; Azpeitia et al. 2021). Notably, most of the horticultural and landscape plants that we enjoy in our public and private gardens are more admired the more out of the ordinary they are.

New methods to create targeted variation, such as genome editing, lead to mutations that are the same or similar to those resulting from naturally occurring or induced variation (Martínez-Fortún et al. 2022). Genome editing methods simply enable the induction of variation in focused locations within a genome. Globally, billions of dollars and millions of hours have been invested to sequence plant genomes, build genomic resources, and uncover the molecular genetics of key traits and features in agriculturally, horticulturally, and culturally important plant species (Marks et al. 2021). Precision breeding methods like genome editing are 1 route to achieve a return on the collective societal investment in plant science. Furthermore, genome editing provides a route to identify and provide innovative solutions for grand challenges in agriculture, such as resilience to global climate change, within our lifetimes. That return promises to include increased food stability and safety, improved nutrition, and access to essential plant sourced vitamins and minerals, as well as reductions in the impacts of agriculture on the environment.

In summary, domestication, diversification, and varietal selection of crops took the collective efforts and will of millions of people over millennia to accomplish. The integration, propagation and assortment of spontaneous and induced mutations have transformed low-yielding, hard to cultivate, and sometimes harmful plants into the highly productive, safe, and nutritious crops we rely on today. This tried, true and safe methodology will only be enhanced by the adoption of modern breeding methods like genome editing.

Advances box

- Genomic sequence variation is the key to diversity in nature and food systems. The scale of sequence variation in plants ranges from single nucleotides to whole-genome duplications.
- Mutations are widespread and frequent in plant genomes, but mutations with agricultural and consumer value are rare.
- The underlying sequence changes that enabled modern crop diversity were not known at the time of domestication and diversification but were safely incorporated into food systems through phenotypic selection and evaluation.
- Diverse sequence changes can lead to convergent phenotypic outcomes. Thus, the potential value of a mutation extends beyond the underlying method that introduces the mutation.
- Targeted breeding technologies like genome editing can enhance the creation and selection of useful variation in crops to meet the evolving needs of farmers, producers, and customers.

Outstanding questions

- Why is variation created through modern breeding methods like genome editing considered to be different from natural and induced mutations, which are currently accepted commercial breeding practices worldwide?
- As large-scale sequencing/phenotyping technologies advance, how can this data help us translate the relationship between genomic and phenotypic variation for genome editing applications?
- How can we better leverage the wealth of large-scale genomic sequencing data to demonstrate the wide range of genomic variability in our food and feed crops and to promote the acceptance of mutations and tools such as genome editing beyond the scientific community?
- Since current regulations for crops limit the potential of targeted mutagenesis as a breeding tool, can we develop an alternative global regulatory framework based on the history of the safe mutations in conventional plant breeding?

## Data Availability

There are no new data associated with this article.
